# Supplementary material for: Knowledge increases informative reporting by the public about urban coyotes
Source: PLoS One. 2025 May 9;20(5):e0307728. doi: 10.1371/journal.pone.0307728 (PMC12063906; doi:10.1371/journal.pone.0307728)
Supplement: S1 Appendix A — The Edmonton Coyote Insights Survey was administered online by the City of Edmonton in Spring 2022. The survey received a total of 5,926 responses. (PDF) [file pone.0307728.s001.pdf]

Appendix A

Insight Survey: Coyote awareness and perception

Survey Responses

The City of Edmonton would like to know how you perceive urban coyotes and their behaviours, how you would react when you see a coyote in various scenarios, and when you would call 311 or expect assistance from the City. The survey will run from April 25, 2022 to May 15, 2022 and should take approximately 12-15 minutes to complete. Feedback from the survey will inform a coyote awareness campaign to help people and coyotes coexist peacefully.

Section 1: General knowledge of coyotes and other wildlife

1. Coyotes are about the size of a border collie, but with fluffy grey-brown coats, long ears and noses and a black tip on their bushy tails. Based on this description and the photo below, have you seen a coyote within the last 12 months anywhere in the City of Edmonton?
   a. Yes (**5,213 participants, 80%**)
   b. No (**646, 11%**)
   c. Not Sure (**67, 1%**) (for NS go to #4)

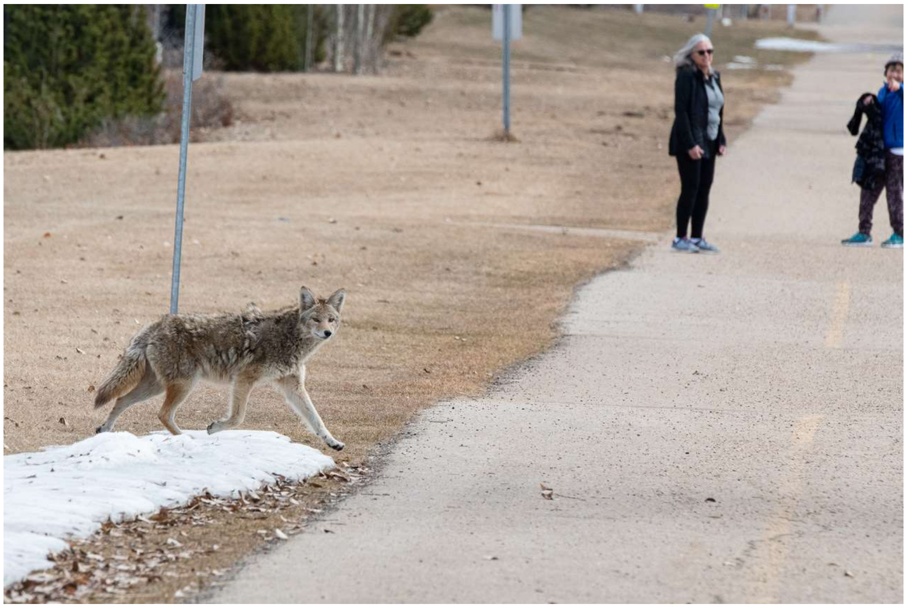

2. In the last 12 months, which kind of encounter(s) have you had with a coyote? (Click all that apply)
    a. I saw a coyote from a car or building. (**3743, 64%**)
    b. I saw a coyote when I was outside of a car or building from a distance of at least 50 metres (approximately 3 city bus lengths). (**2574, 44%**)
    c. I saw a coyote when I was outside and it was closer than 50 metres. (**2618, 45%**)
    d. A coyote approached me while I was walking, jogging or cycling. (**604, 10%**)
    e. A coyote tried to bite me or my pet. (**79, 1%**)
    f. A coyote bit or killed my pet (please describe below). (**50, 1%**)
    g. Have had no encounter with a coyote in the past 12 months (**638, 11%**)

3. In the last 12 months, which of these animals have you seen in your neighbourhood (e.g., Strathcona)? (Check all that apply)
    a. Birds on the ground (**5311, 91%**)
    b. Jack rabbits (prefer open areas) or snowshoe hares (prefer wooded areas) (**5663, 97%**)

c. Coyotes (**4672, 80%**)

d. Deer (**821, 14%**)

e. One or more dogs off leash (**3825, 65%**)

f. One or more cats off leash (domestic or feral) (**4663, 80%**)

g. Tree squirrels (**4878, 83%**)

h. Small rodents (such as mice and voles) (**2687, 46%**)

i. Other: _______ (**628, 11%**)

j. None of the above (**15, 0%**)

4. In the last 12 months, have you seen any of the following items outside of fenced yards in your neighbourhood (e.g., Strathcona) and greenspaces (e.g., parks and ravines)? (Check all that apply)

	In your neighbourhood, outside of fenced yards	Parks, Greenspace, or River Valley	Not Seen
Bird or squirrel feeders hung above the ground	**2700, 46%**	**1696, 29%**	**2533, 43%**
Bird seed or peanuts on the ground	**2101, 35%**	**1249, 21%**	**3198, 54%**
Alfalfa pellets or hay	**173, 3%**	**203, 3%**	**5586, 94%**
Pet food on the ground	**394, 7%**	**130, 2%**	**5461, 92%**
Meat scraps or bones	**1162, 20%**	**464, 8%**	**4580, 77%**
Unsecured garbage (e.g., bags or overflowing bins)	**3789, 64%**	**1458, 25%**	**1857, 31%**
Unsecured compost (e.g., piles or bins with 3 sides)	**882, 15%**	**177, 3%**	**5004, 84%**
Fallen fruit on the ground from fruit trees	**3259, 55%**	**1297, 22%**	**2403, 41%**

If you have seen other, similar items, list them here ______________________________

5. Thinking of coyotes that regularly access human sources of food in urban areas, to what extent would you agree with each of the following statements?

	Strongly agree	Agree	Neutral	Disagree	Strongly disagree
They are more likely to survive and reproduce.	**2479**<br>**42%**	**2038**<br>**34%**	**982**<br>**17%**	**308**<br>**5%**	**119**<br>**2%**
They lose their fear of people.	**3121**<br>**53%**	**2076**<br>**35%**	**406**<br>**7%**	**235**<br>**4%**	**88**<br>**1%**
They become dependent on human sources of food.	**2106**<br>**36%**	**2,367**<br>**40%**	**911**<br>**15%**	**421**<br>**7%**	**121**<br>**2%**
They are more likely to carry diseases, including some that people can get.	**1030**<br>**17%**	**1380**<br>**23%**	**2427**<br>**41%**	**743**<br>**13%**	**6346**<br>**6%**
They are more likely to be aggressive towards people or pets.	**2086**<br>**35%**	**2109**<br>**36%**	**1168**<br>**20%**	**411**<br>**7%**	**152**<br>**3%**
They are more likely to den nearby.	**2289**	**2432**	**931**	**212**	**62**

	**39%**	**41%**	**16%**	**4%**	**1%**
They are more likely to be killed by wildlife managers to protect the public.	**1900**<br>**32%**	**1718**<br>**29%**	**1540**<br>**26%**	**447**<br>**8%**	**321**<br>**5%**

Section 2: Attitudes toward coyotes

6. To what extent do you **agree** with each of the following?

	**Strongly agree**	**Agree**	**Neutral**	**Disagree**	**Strongly disagree**
Coyotes provide ecological benefits in Edmonton.	**1646**<br>**28%**	**2035**<br>**34%**	**1353**<br>**23%**	**521**<br>**9%**	**370**<br>**6%**
Coyotes injure and kill too many pets in Edmonton.	**603**<br>**10%**	**1077**<br>**18%**	**2242**<br>**38%**	**1172**<br>**20%**	**831**<br>**14%**
The presence of coyotes in Edmonton is a sign of a healthy environment.	**953**<br>**16%**	**1791**<br>**30%**	**1794**<br>**30%**	**935**<br>**16%**	**452**<br>**8%**
There are too many coyotes in Edmonton.	**952**<br>**16%**	**1112**<br>**19%**	**2112**<br>**36%**	**935**<br>**16%**	**814**<br>**14%**

Coyotes frequently pose a threat to people in Edmonton.	**447**<br>**8%**	**1060**<br>**18%**	**1336**<br>**23%**	**1668**<br>**28%**	**1414**<br>**24%**
If a coyote bites a human, the City of Edmonton should identify and kill the animal.	**1817**<br>**31%**	**1357**<br>**23%**	**1201**<br>**20%**	**909**<br>**15%**	**641**<br>**11%**
Coyotes have a right to exist in my neighbourhood if they are not harming people.	**1900**<br>**32%**	**1682**<br>**28%**	**977**<br>**16%**	**761**<br>**13%**	**605**<br>**10%**

7. Given the presence of coyotes in Edmonton, how do you **feel** about each of the following?

**I am concerned about…**	**Strongly agree**	**Agree**	**Neutral**	**Disagree**	**Strongly disagree**	**N/A**
…my own personal health or safety.	**592**<br>**10%**	**1154**<br>**19%**	**870**<br>**15%**	**1324**<br>**22%**	**1955**<br>**33%**	**30**<br>**1%**
…my children's health or safety.	**953**<br>**16%**	**834**<br>**14%**	**556**<br>**9%**	**621**<br>**10%**	**845**<br>**14%**	**2116**<br>**36%**
… my pet's health or safety.	**1339**<br>**23%**	**1215**<br>**21%**	**532**<br>**9%**	**586**<br>**10%**	**745**<br>**13%**	**1508**<br>**25%**

… the spread of diseases carried by coyotes.	**779 13%**	**1462 25%**	**1511 26%**	**1079 18%**	**1055 18%**	**39 1%**

8. Which of the following coyote behaviours would concern you and / or cause you to report to 311?

	**Yes**	**No**	**Not Sure**
A coyote is in a residential yard during the day.	**3236, 55%**	**1932, 33%**	**757, 13%**
A coyote is in a school yard at dawn or dusk.	**2077, 35%**	**3149, 53%**	**699, 12%**
Coyotes follow me within 50 m in a natural area.	**1985, 34%**	**3029, 51%**	**911, 15%**
A coyote follows me within 50 m in my neighbourhood.	**3605, 61%**	**1673, 28%**	**647, 11%**
A coyote is hunting small rodents in a field near houses.	**506, 9%**	**4912, 83%**	**507, 9%**
A coyote in a residential area does not run away after I	**3870,**	**1073,**	**982,**

attempt to intimidate it by shouting and throwing sticks.	**65%**	**18%**	**17%**
A coyote is eating food that appears to be left out for it intentionally on the edge of a natural area.	**3940, 66%**	**1329, 22%**	**656, 11%**
I hear coyotes howling and yipping from my home.	**624, 11%**	**4945, 83%**	**356, 6%**
A coyote approaches me and my large dog (on a leash) as we walk in my neighbourhood.	**3533, 60%**	**1308, 22%**	**1084, 18%**
I see a coyote that appears sick or injured to the point where it can't move.	**5320, 90%**	**405, 7%**	**200, 3%**
I see a coyote with other coyotes or pups at a distance.	**1215, 21%**	**4123, 70%**	**587, 10%**
I find a coyote den or see very young pups.	**1959, 33%**	**3095, 52%**	**871, 15%**

Section 3: Situations involving coyotes

We will describe two different situations involving you and a coyote. Think about what each situation would be like for you. Then identify the response closest to your feelings and opinions.

Scenario #1: Imagine you are walking alone along a **trail in a park, greenspace or the River**

Valley in Edmonton during the day and a coyote crosses the trail 15 m (one bus length) ahead of you and stops to look at you.

9. To what extent would you agree with the following statements?

I would …	Strongly agree	Agree	Neutral	Disagree	Strongly disagree	Unsure
… feel comfortable with this	**1084**	**1765**	**799**	**1455**	**773**	**49**
scenario.	**18%**	**30%**	**13%**	**25%**	**13%**	**1%**
… think this scenario should	**401**	**602**	**991**	**1697**	**2169**	**65**
not occur in Edmonton.	**7%**	**10%**	**17%**	**29%**	**37%**	**1%**
… run away.	**130**	**288**	**815**	**1665**	**2849**	**178**
	**2%**	**5%**	**14%**	**28%**	**48%**	**3%**
… stand tall, talk loudly and	**1548**	**2235**	**980**	**640**	**337**	**185**
try to back away slowly.	**26%**	**38%**	**17%**	**11%**	**6%**	**3%**
… stand still and do nothing	**770**	**2192**	**1101**	**1069**	**611**	**82**
until the animal leaves.	**13%**	**37%**	**19%**	**18%**	**10%**	**3%**
… continue to walk and	**501**	**1369**	**901**	**1653**	**1363**	**138**
ignore the coyote.	**8%**	**23%**	**15%**	**28%**	**23%**	**2%**
… approach the coyote while	**616**	**1309**	**921**	**1257**	**1640**	**182**
throwing objects towards it,	**10%**	**22%**	**16%**	**21%**	**28%**	**3%**

banging a stick on nearby trees and otherwise trying to intimidate it.						
… notify the City via the 311 phone line or app.	**723**<br>**12%**	**778**<br>**13%**	**1112**<br>**19%**	**1220**<br>**21%**	**1685**<br>**28%**	**407**<br>**7%**

10. To what extent would you agree with the following statements?

**Management Action**	**Strongly agree**	**Agree**	**Neither**	**Disagree**	**Strongly disagree**	**Unsure**
311/City of Edmonton Park Rangers need to be made aware of this incident for tracking purposes only.	**847**<br>**14%**	**1580**<br>**27%**	**1175**<br>**20%**	**1083**<br>**18%**	**803**<br>**14%**	**438**<br>**7%**
311/City of Edmonton Park Rangers do not need to be notified of this incident.	**1038**<br>**18%**	**1552,**<br>**26%**	**1093**<br>**18%**	**1103**<br>**19%**	**750**<br>**13%**	**390**<br>**7%**
Public needs to be educated about safety around coyotes in this area.	**2904**<br>**49%**	**2331**<br>**39%**	**419**<br>**7%**	**160**<br>**3%**	**78**<br>**1%**	**34**<br>**1%**
Park Rangers should look for	**470**	**744**	**1331**	**1606**	**1493**	**282**

Management Action	Strongly agree	Agree	Neither	Disagree	Strongly disagree	Unsure
311/City of Edmonton Park Rangers need to be made aware of this incident for tracking purposes only.	**847**<br>**14%**	**1580**<br>**27%**	**1175**<br>**20%**	**1083**<br>**18%**	**803**<br>**14%**	**438**<br>**7%**
311/City of Edmonton Park Rangers do not need to be notified of this incident.	**1038**<br>**18%**	**1552,**<br>**26%**	**1093**<br>**18%**	**1103**<br>**19%**	**750**<br>**13%**	**390**<br>**7%**
the coyote and frighten it from the area.	**8%**	**13%**	**22%**	**27%**	**25%**	**5%**
Attempt to capture and relocate the coyote.	**583**<br>**10%**	**695**<br>**12%**	**873**<br>**15%**	**1414**<br>**24%**	**2134**<br>**36%**	**227**<br>**4%**
Attempt to capture and kill the coyote.	**5271**<br>**5%**	**140**<br>**2%**	**365**<br>**6%**	**704**<br>**12%**	**4340**<br>**73%**	**106**<br>**2%**

Scenario #2: Now imagine you are out walking alone in your **neighbourhood** during the day and see a coyote in the alleyway approaching yards. You know from your community social media site that several others have seen a coyote recently in the same area.

11. How would you respond to the following statements?

I would …	Strongly agree	Agree	Neutral	Disagree	Strongly disagree	Unsure
… feel comfortable with this scenario.	**640**<br>**11%**	**1248**<br>**21%**	**955**<br>**16%**	**1843**<br>**31%**	**1199**<br>**20%**	**41**<br>**1%**
… think this scenario should not occur in Edmonton.	**794**<br>**13%**	**1179**<br>**20%**	**1060**<br>**18%**	**1518**<br>**26%**	**1309**<br>**22%**	**66**<br>**1%**
… run away.	**121**<br>**2%**	**313**<br>**5%**	**823**<br>**14%**	**1658**<br>**28%**	**2807**<br>**47%**	**204**<br>**3%**
… stand tall, talk loudly and try to back away slowly.	**1092**<br>**18%**	**2452**<br>**41%**	**960**<br>**16%**	**719**<br>**12%**	**493**<br>**8%**	**210**<br>**4%**
… stand still and do nothing until the animal leaves.	**354**<br>**6%**	**1927**<br>**33%**	**1310**<br>**22%**	**1333**<br>**22%**	**774**<br>**13%**	**228**<br>**4%**
… continue to walk and ignore the coyote.	**459**<br>**8%**	**1416**<br>**24%**	**909**<br>**15%**	**1620**<br>**27%**	**1368**<br>**23%**	**154**<br>**3%**
… approach the coyote while throwing objects towards it, banging a stick on nearby trees and otherwise trying to intimidate it.	**659**<br>**11%**	**1391**<br>**23%**	**782**<br>**13%**	**1211**<br>**20%**	**1689**<br>**29%**	**194**<br>**3%**

… notify the City via the 311 phone line or app.	**1444**<br>**24%**	**1582**<br>**27%**	**881**<br>**15%**	**771**<br>**13%**	**884**<br>**15%**	**364**<br>**6%**

12. To what extent would you agree with the following statements?

**Management Action**	Strongly agree	Agree	Neither	Disagree	Strongly disagree	Unsure
311/City of Edmonton Park Rangers need to be made aware of this incident for tracking purposes only.	**1450**<br>**24%**	**2235**<br>**38%**	**825**<br>**14%**	**641**<br>**11%**	**481**<br>**8%**	**294**<br>**5%**
311/City of Edmonton Park Rangers do not need to be notified of this incident.	**515**<br>**9%**	**816**<br>**14%**	**1044**<br>**18%**	**1739**<br>**29%**	**1476**<br>**25%**	**336**<br>**6%**
Public needs to be educated about safety around coyotes in this area.	**3285**<br>**55%**	**2070**<br>**35%**	**368**<br>**6%**	**82**<br>**1%**	**76**<br>**1%**	**45**<br>**1%**
Park Rangers should look for the coyote and frighten it from the area.	**903**<br>**15%**	**1577**<br>**27%**	**1246**<br>**21%**	**1036**<br>**17%**	**882**<br>**15%**	**282**<br>**5%**

**Management Action**	Strongly agree	Agree	Neither	Disagree	Strongly disagree	Unsure
311/City of Edmonton Park Rangers need to be made aware of this incident for tracking purposes only.	**1450**<br>**24%**	**2235**<br>**38%**	**825**<br>**14%**	**641**<br>**11%**	**481**<br>**8%**	**294**<br>**5%**
Attempt to capture and relocate the coyote.	**1081**<br>**18%**	**1349**<br>**23%**	**836**<br>**14%**	**1062**<br>**18%**	**1349**<br>**23%**	**249**<br>**4%**
Attempt to capture and kill the coyote.	**321**<br>**5%**	**167**<br>**3%**	**378**<br>**6%**	**793**<br>**13%**	**4119**<br>**70%**	**148**<br>**2%**

Section 4: Perceptions and knowledge of the City of Edmonton's Coyote Response Strategy

13. 'Hazing' (also called 'aversive conditioning' or 'behaviour conditioning') is a method that makes use of deterrents to move an animal out of an area or discourage an undesirable behaviour or activity (example: approaching coyotes while shouting and throwing tennis balls weighted with sand or throwing sticks and stones near the coyote). Before today had you heard of hazing/aversive conditioning?
    a. Yes **(3691, 62%)**
    b. No (**2009, 34%**)
    c. Not Sure (**226, 4%**)

14. What would your comfort level be using hazing/aversive conditioning techniques to deter coyotes in a situation that could endanger pets or vulnerable people?

1 - Very uncomfortable (**700, 12%**)

2 - Uncomfortable (**943, 16%**)

3 - Neutral (**1101, 19%**)

4 - Comfortable (**1985, 33%**)

5 - Very comfortable (**1153, 19%**)

0 - N/A (**44, 1%**)

15. Have you ever called 311 (the City of Edmonton) to report a coyote?
    a. Yes (**642, 11%**)
    b. No (**5242, 88%**)
    c. Not Sure (**42, 1%**)

a. [If yes to 15] What were the circumstances or coyote behaviours witnessed?

   ____________________ (open)

16. Do you have anything else you would like to share with us about coyotes? Open Optional

DEMOGRAPHICS

Answering the following demographic questions will help us ensure we are hearing from a variety of perspectives and providing inclusive information later.

1. Do you currently?

    Live in Edmonton (**5869, 99%**)

    Live in surrounding areas (**43, 1%**)

    I prefer not to answer (**14, 0%**)

2. [IF LIVE IN EDMONTON] Which neighbourhood do you live in?

    (Dropdown list including don't know/prefer not to answer)

**Neighbourhood**	**%**	**Count**
Abbottsfield	**0.1**	**7**
Albany	**0.2**	**15**
Alberta Avenue	**0.5**	**32**
Alberta Park Industrial	**0**	**1**
Aldergrove	**0.5**	**33**
Allard	**0.3**	**18**
Allendale	**0.4**	**29**
Ambleside	**0.3**	**18**
Anthony Henday Big Lake	**0**	**1**
Anthony Henday Castledowns	**0.1**	**6**
Anthony Henday Clareview	**0**	**1**
Anthony Henday South	**0**	**1**

Anthony Henday South East	**0**	**1**
Anthony Henday South West	**0.1**	**4**
Anthony Henday Terwillegar	**0.1**	**4**
Argyll	**0.2**	**12**
Aspen Gardens	**0.4**	**26**
Athlone	**0.2**	**16**
Avonmore	**0.8**	**51**
Balwin	**0.2**	**11**
Bannerman	**0.2**	**15**
Baranow	**0.1**	**4**
Baturyn	**0.5**	**33**
Beacon Heights	**0.2**	**11**
Bearspaw	**0.4**	**26**
Beaumaris	**0.4**	**24**
Belgravia	**0.5**	**32**
Belle Rive	**0.1**	**7**
Bellevue	**0.3**	**17**
Belmead	**0.3**	**22**
Belmont	**0.2**	**14**
Belvedere	**0.2**	**13**
Bergman	**0.1**	**9**
Beverly Heights	**0.4**	**25**
Bisset	**0.2**	**13**

Blackburne	**0.3**	**21**
Blackmud Creek	**0.3**	**17**
Blackmud Creek Ravine	**0.1**	**6**
Blue Quill	**0.5**	**32**
Blue Quill Estates	**0.1**	**6**
Bonnie Doon	**0.9**	**58**
Boyle Street	**0.2**	**13**
Brander Gardens	**0.3**	**20**
Breckenridge Greens	**0.2**	**11**
Brintnell	**0.3**	**18**
Britannia Youngstown	**0.3**	**22**
Brookside	**0.3**	**18**
Bulyea Heights	**0.4**	**28**
Caernarvon	**0.2**	**16**
Calder	**0.3**	**19**
Callaghan	**0.3**	**18**
Callingwood North	**0.4**	**23**
Callingwood South	**0.2**	**13**
Cameron Heights	**0.2**	**12**
Canon Ridge	**0.1**	**7**
Canora	**0.1**	**6**
Canossa	**0.2**	**10**
Capilano	**0.6**	**39**

Carlisle	**0.2**	**15**
Carlton	**0.4**	**23**
Carter Crest	**0.1**	**9**
Casselman	**0.2**	**12**
Cavanagh	**0**	**3**
Central McDougall	**0.1**	**7**
Chambery	**0.1**	**7**
Chappelle Area	**0.4**	**23**
Charlesworth	**0.2**	**14**
Clareview Town Centre	**0.2**	**15**
Cloverdale	**0.4**	**23**
CPR Irvine	**0**	**2**
Crawford Plains	**0.3**	**17**
Crestwood	**0.4**	**26**
Cromdale	**0.2**	**12**
Crystallina Nera East	**0**	**3**
Crystallina Nera West	**0.1**	**4**
Cumberland	**0.4**	**28**
Cy Becker	**0.2**	**13**
Daly Grove	**0.1**	**8**
Dechene	**0.2**	**10**
Delton	**0.2**	**16**
Delwood	**0.5**	**34**

Desrochers Area	**0.1**	**6**
Donsdale	**0.1**	**6**
Dovercourt	**0.3**	**21**
Downtown	**1.1**	**68**
Duggan	**0.7**	**48**
Dunluce	**0.4**	**23**
Eastwood	**0.1**	**7**
Eaux Claires	**0.2**	**16**
Ebbers	**0**	**2**
Edgemont	**0.4**	**24**
Edmonton Northlands	**0**	**1**
Ekota	**0.2**	**16**
Ellerslie	**0.2**	**15**
Elmwood	**0.2**	**12**
Elmwood Park	**0.1**	**4**
Elsinore	**0.1**	**7**
Empire Park	**0.2**	**16**
Ermineskin	**0.5**	**30**
Evansdale	**0.2**	**14**
Evergreen	**0.1**	**4**
Falconer Heights	**0.1**	**8**
Forest Heights	**0.6**	**37**
Fraser	**0.4**	**26**

Fulton Place	**0.5**	**33**
Gainer Industrial	**0**	**1**
Gariepy	**0.3**	**19**
Garneau	**0.6**	**41**
Glastonbury	**0.5**	**35**
Glengarry	**0.3**	**19**
Glenora	**0.6**	**38**
Glenridding Area	**0.2**	**11**
Glenwood	**0.2**	**14**
Gold Bar	**0.6**	**38**
Grandview Heights	**0.2**	**14**
Granville	**0.1**	**7**
Graydon Hill	**0.1**	**5**
Greenfield	**0.7**	**45**
Greenview	**0.4**	**25**
Griesbach	**0.7**	**44**
Grovenor	**0.4**	**29**
Haddow	**0.6**	**36**
Hairsine	**0.2**	**11**
Hawks Ridge	**0.2**	**10**
Hays Ridge Area	**0**	**1**
Hazeldean	**0.4**	**25**
Henderson Estates	**0.3**	**18**

Heritage Valley Area	**0.1**	**7**
Heritage Valley Town Centre Area	**0**	**1**
High Park	**0.2**	**10**
Highlands	**1.1**	**71**
Hillview	**0.3**	**19**
Hodgson	**0.1**	**8**
Hollick-Kenyon	**0.3**	**20**
Holyrood	**0.4**	**29**
Homesteader	**0.2**	**13**
Hudson	**0.2**	**14**
I don't know	**0.1**	**5**
I prefer not to answer	**0.8**	**51**
Idylwylde	**0.2**	**16**
Inglewood	**0.6**	**41**
Jackson Heights	**0.3**	**18**
Jamieson Place	**0.3**	**20**
Jasper Park	**0.1**	**6**
Kameyosek	**0.2**	**11**
Keheewin	**0.2**	**15**
Kenilworth	**0.3**	**21**
Kensington	**0.4**	**29**
Kernohan	**0.2**	**14**

Keswick Area	**0.2**	**11**
Kildare	**0.2**	**12**
Kilkenny	**0.4**	**25**
Killarney	**0.3**	**18**
King Edward Park	**0.8**	**49**
Kiniski Gardens	**0.4**	**23**
Kirkness	**0.3**	**18**
Klarvatten	**0.2**	**16**
La Perle	**0.7**	**46**
Lago Lindo	**0.2**	**16**
Lansdowne	**0.3**	**17**
Larkspur	**0.3**	**18**
Lauderdale	**0.2**	**14**
Laurel	**0.3**	**17**
Laurier Heights	**0.6**	**39**
Lee Ridge	**0.2**	**10**
Leger	**0.1**	**8**
Lendrum Place	**0.3**	**21**
Lewis Farms Industrial	**0.1**	**6**
Lorelei	**0.2**	**13**
Lymburn	**0.4**	**26**
Lynnwood	**0.4**	**27**
MacEwan	**0.5**	**32**

Mactaggart	**0.3**	**19**
Magrath Heights	**0.2**	**15**
Malmo Plains	**0.5**	**31**
Maple	**0.1**	**7**
Maple Ridge	**0**	**3**
Maple Ridge Industrial	**0**	**1**
Matt Berry	**0.2**	**15**
Mayfield	**0.2**	**14**
Mayliewan	**0.2**	**13**
McCauley	**0.2**	**14**
McConachie Area	**0.5**	**30**
McKernan	**0.1**	**9**
McLeod	**0.2**	**16**
McQueen	**0.2**	**15**
Meadowlark Park	**0.3**	**17**
Meadows Area	**0.3**	**22**
Menisa	**0.2**	**14**
Meyokumin	**0.2**	**13**
Meyonohk	**0.1**	**9**
Michaels Park	**0.1**	**8**
Mill Creek Ravine North	**0.2**	**13**
Mill Creek Ravine South	**0.1**	**6**
Mill Woods Golf Course	**0.1**	**4**

Mill Woods Park	**0.1**	**5**
Mill Woods Town Centre	**0.2**	**13**
Miller	**0.2**	**13**
Minchau	**0.2**	**12**
Montrose	**0.2**	**16**
Newton	**0.2**	**16**
North Glenora	**0.5**	**34**
Northmount	**0.1**	**8**
Norwester Industrial	**0**	**2**
Ogilvie Ridge	**0.1**	**9**
Oleskiw	**0.4**	**23**
Oliver	**2.4**	**157**
Ormsby Place	**0.4**	**25**
Other	**0.2**	**10**
Ottewell	**1.2**	**77**
Overlanders	**0.2**	**10**
Oxford	**0.2**	**15**
Ozerna	**0.2**	**12**
Paisley	**0.1**	**7**
Parkallen	**0.6**	**41**
Parkdale	**0.2**	**15**
Parkview	**0.8**	**49**
Parsons Industrial	**0**	**1**

Patricia Heights	**0.2**	**14**
Pleasantview	**0.5**	**34**
Pollard Meadows	**0.1**	**9**
Potter Greens	**0.2**	**11**
Prince Charles	**0.1**	**7**
Prince Rupert	**0.2**	**10**
Queen Alexandra	**0.5**	**31**
Queen Mary Park	**0.7**	**44**
Quesnell Heights	**0**	**2**
Ramsay Heights	**0.5**	**30**
Rapperswill	**0.3**	**17**
Rhatigan Ridge	**0.3**	**21**
Richfield	**0.2**	**11**
Richford	**0**	**3**
Rideau Park	**0.3**	**18**
Rio Terrace	**0.3**	**20**
Ritchie	**0.9**	**56**
River Valley Hermitage	**0**	**1**
River Valley Lessard North	**0**	**1**
River Valley Oleskiw	**0**	**1**
River Valley Riverside	**0**	**1**
River Valley Terwillegar	**0.1**	**8**
River Valley Victoria	**0**	**2**

River Valley Whitemud	**0**	**2**
River Valley Windermere	**0**	**1**
Riverdale	**0.7**	**46**
Riverview Area	**0**	**3**
Rosenthal	**0.1**	**9**
Rossdale	**0.2**	**12**
Rosslyn	**0.1**	**9**
Royal Gardens	**0.4**	**26**
Rundle Heights	**0.2**	**10**
Rural North East Horse Hill	**0**	**3**
Rural North East South Sturgeon	**0**	**1**
Rutherford	**1.3**	**86**
Sakaw	**0.3**	**18**
Satoo	**0.4**	**24**
Schonsee	**0.1**	**7**
Secord	**0.3**	**19**
Sherbrooke	**0.2**	**11**
Sherwood	**0.1**	**4**
Sifton Park	**0.1**	**7**
Silver Berry	**0.3**	**18**
Skyrattler	**0.3**	**18**
South Edmonton Common	**0**	**2**

South Terwillegar	**0.6**	**36**
Spruce Avenue	**0.1**	**8**
Starling	**0.1**	**7**
Steinhauer	**0.4**	**26**
Stewart Greens	**0**	**2**
Strathcona	**1.5**	**99**
Strathearn	**0.6**	**36**
Suder Greens	**0.1**	**7**
Summerlea	**0.1**	**4**
Summerside	**0.4**	**28**
Sweet Grass	**0.2**	**14**
Tamarack	**0.1**	**8**
Tawa	**0.2**	**10**
Terra Losa	**0.2**	**12**
Terrace Heights	**0.6**	**37**
Terwillegar Towne	**0.8**	**51**
The Hamptons	**0.4**	**26**
The Orchards At Ellerslie	**0.2**	**14**
Thorncliff	**0.2**	**11**
Tipaskan	**0.2**	**11**
Trumpeter Area	**0.2**	**13**
Tweddle Place	**0.2**	**10**
Twin Brooks	**0.7**	**43**

University of Alberta Farm	**0**	**1**
Virginia Park	**0.1**	**9**
Walker	**0.3**	**20**
Webber Greens	**0.2**	**14**
Wedgewood Heights	**0.1**	**6**
Weinlos	**0.1**	**9**
Wellington	**0.3**	**17**
West Jasper Place	**0.1**	**8**
West Meadowlark Park	**0.2**	**10**
Westbrook Estates	**0**	**3**
Westmount	**1.2**	**75**
Westridge	**0.2**	**11**
Westview Village	**0.1**	**4**
Westwood	**0.2**	**14**
Whitemud Creek Ravine		
North	**0**	**1**
Whitemud Creek Ravine		
South	**0**	**2**
Wild Rose	**0.2**	**14**
Windermere	**0.6**	**36**
Windermere Area	**0.2**	**11**
Windsor Park	**0.2**	**15**

Winterburn Industrial Area		
West	**0**	**1**
Woodcroft	**0.2**	**14**
York	**0.4**	**24**

3. [IF Edmonton Q1] What are the first 3 digits of your postal code?

    a. List

    b. I don't know

    c. I prefer not to answer

Postal Code	Percent	Frequency
T5A	**2.7**	**172**
T5B	**1.6**	**102**
T5C	**1.4**	**89**
T5E	**2.5**	**164**
T5G	**1.2**	**76**
T5H	**2.3**	**149**
T5J	**0.6**	**37**
T5K	**2.9**	**186**
T5L	**1.4**	**92**
T5M	**2.3**	**146**
T5N	**2.2**	**141**
T5P	**1.2**	**79**
T5R	**3**	**197**

T5S	**0.6**	**38**
T5T	**5.4**	**346**
T5V	**0**	**3**
T5W	**2.1**	**137**
T5X	**2.6**	**171**
T5Y	**2.9**	**190**
T5Z	**1.4**	**88**
T6A	**2.9**	**190**
T6B	**1.7**	**107**
T6C	**4**	**261**
T6E	**3.4**	**217**
T6G	**1.3**	**85**
T6H	**4.1**	**263**
T6J	**5.6**	**365**
T6K	**1.9**	**123**
T6L	**3.3**	**216**
T6M	**2.2**	**139**
T6N	**0**	**2**
T6P	**0.1**	**4**
T6R	**4.3**	**277**
T6T	**1.5**	**96**
T6V	**1.4**	**92**
T6W	**5**	**325**

T6X	**1.4**	**90**
T6Y	**0**	**1**
I don't know	**0.1**	**6**
I prefer not to answer	**1.5**	**96**

4. What type of a dwelling do you live in?
   a. Single detached home (**4207, 71%**)
   b. Townhouse, duplex, or fourplex (**761, 13%**)
   c. Condo/apartment (**822, 14%**)
   d. Other (Please specify) (**43, 1%**)
   e. I prefer not to answer (**93, 2%**)

5. Do you have a yard facing or back onto a park or natural area (e.g.,ravine, river valley, utility corridor)?
   a. Yes (**1699, 29%**)
   b. No (**4146, 70%**)
   c. I don’t know (**30, 1%**)
   d. I prefer not to answer (**51, 1%**)

6. Which of the following describes your household? (Please check all that apply)
   a. We have children under 13 in the household (**1113, 19%**)
   b. We have people 13-64 in the household (**3702, 62%**)

c. We have people 65 and above in the household (**1650, 28%**)
d. Other (Please specify): (**230, 4%**)
e. I prefer not to answer (**268, 5%**)

7. Which of the following describes pets in your home? (choose all that apply)
   a. I have one or more indoor cats (**1350, 23%**)
   b. I have one or more outdoor cats (that may sometimes escape my yard) (**320, 5%**)
   c. I have one or more small dogs (smaller than a border collie) (**1236, 21%**)
   d. I have one or more large dogs (as large or larger than a border collie) (**1298, 22%**)
   e. My dogs are sometimes off leash outside my yard (in or outside designated areas) (**759, 13%**)
   f. I have other outdoor pets named below. (**34, 1%**)
   g. Other ______________________ (**228, 4%**)
   h. I have no pets (**2233, 38%**)
   i. I prefer not to answer (**146, 2%**)

8. Are you?

   Under 18 years old (**5, 0%**)

   18-24 years old (**89, 2%**)

   25-34 years old (**683, 12%**)

   35-44 years old (**1210, 20%**)

   45-54 years old (**1085, 18%**)

   55-64 years old (**1264, 21%**)

65 to 74 years old (**1096, 18%**)

75+ years old (**228, 4%**)

I prefer not to answer (**266, 4%**)

9. What is your gender? Choose all that apply.

    Woman (**3270, 55%**)

    Man (**2179, 37%**)

    Non-binary (**57, 1%**)

    Transgender (**27, 0%**)

    Two-Spirit (**23, 0%**)

    Another gender not listed above (**24, 0%**)

    I prefer not to answer (**410, 7%**)

10. Are you a member of any of the following groups? (Please check all that apply)

    a. Racialized / visible minority (**356, 6%**)

    b. Persons with disabilities (**407, 7%**)

    c. Indigenous (**134, 2%**)

    d. New to Canada (**48, 1%**)

    e. None of these (**4263, 72%**)

    f. Other (Please specify): (**193, 3%**)

    g. I prefer not to answer (**649, 11%**)

11. What is the primary language spoken in your household?

English (**5501, 93%**)

French (**36, 1%**)

Arabic (**4, 0%**)

Cantonese (**17, 0%**)

German (**8, 0%**)

Mandarin (**6, 0%**)

Punjabi (**10, 0%**)

Spanish (**17, 0%**)

Tagalog (Pilipino, Filipino) (**11, 0%**)

Ukrainian (**14, 0%**)

Other (Specify) (**72, 1%**)

I prefer not to answer (**230, 4%**)

12. What is the highest level of education you have completed?

Elementary/grade school graduate (**28, 0%**)

High school graduate (**577, 10%**)

College / technical school graduate (**1596, 27%**)

University undergraduate degree (**1955, 33%**)

Post-graduate degree (e.g. Masters, PhD) (**1094, 18%**)

Professional school graduate (e.g. medicine, dentistry, veterinary medicine, optometry) (**239, 4%**)

I prefer not to answer (**437, 7%**)

Thank you for your feedback. This information will be used to inform a coyote awareness campaign. For more information on urban coyotes, visit edmonton.ca/coyotes or edmontonurbancoyotes.ca.
