# Supplementary material for: Knowledge increases informative reporting by the public about urban coyotes
Source: PLoS One. 2025 May 9;20(5):e0307728. doi: 10.1371/journal.pone.0307728 (PMC12063906; doi:10.1371/journal.pone.0307728)
Supplement: Attachment — Submitted filename: Response to Reviewers.docx [file pone.0307728.s003.docx]

21 November 2024

Dear Editor,

Thank you for the invitation to revise and resubmit the manuscript “Knowledge of risks associated with food-conditioned by coyotes increases the likelihood of informative reporting by the public”. We are grateful for the helpful feedback from you and several reviewers and have adjusted the manuscript according to these suggestions. We provide a summary of those changes below and detail the changes on the following pages.

Our main changes were to simplify the title, refine keywords, improve figure image quality, correct typos and unclear language, and ensure the document complies with PLOS ONE formatting guidelines. We also edited the abstract to improve clarity and ensure it follows the IMRaD framework, added details and clarified phrasing about some background information, the goal of our study, and how and why we employed path analysis, and explained how we handled missing data. We elaborated on the limitations of our study, the implications our limitations have on the interpretation and application of our results, and how our approach can be applied to other human-wildlife conflict management contexts. We further explained potential future work that could stem from this path analysis. Finally, we removed funding information from the Acknowledgements section and amended our funding statement.

At the end of this letter, we copied the reviewers’ comments (in black). We followed each comment with a description of how we addressed it (in blue + a screenshot showing the changes). Thanks for your attention to our manuscript; we look forward to your further news.

Sincerely,

Abby L. Keller

Amended Funding Statement:

This research was supported by a Natural Sciences and Engineering Research Council (NSERC) Discovery Grant (RES0061126) awarded to CCSC and an Alberta Graduate Excellence Scholarship awarded to ALK. The funders had no role in study design, data collection or analysis, manuscript preparation, or the decision to publish. There was no additional external funding received for this study.

**Reviewer #1:**
1. The title of this paper needs revised

We simplified the title while retaining key information as follows:

2. Abstract is not well written (IMRaD is a standard format for writing abstracts in an orderly manner ...such as i. Introduction, ii Methodology, iii Results and iv Discussions). A summary of the result is missing from this abstract

Our previous abstract followed this order, but we adjusted its content to illustrate the key results of our path model more clearly.

3. Some Keywords are not refined enough (e.g The human body posture estimation)

We refined the keywords to increase their relevance.
4. The introduction can be enriched by briefly describe the state-of-the-art in the title of this study and provide more recently related references to support foundation of this studies to better context for the current research. I will recommend citing recently published related papers;
a. Statistical Analysis of Stakeholders Perception on Adoption of AI/ML in Sustainable Agricultural Practices in Rural Development. In X. S. Yang, S. Sherratt, N. Dey, & A. (. Joshi (Ed.), Proceedings of Ninth International Congress on Information and Communication Technology. ICICT 2024 2024. Lecture Notes in Networks and Systems. 1003. Springer, Singapore. doi:<https://doi.org/10.1007/978-981-97-3302-6_11>
b. Crime Prediction Using Twitter Sentiments and Crime Data. Informatica, 48(6), 35–42. doi:<https://doi.org/10.31449/inf.v48i6.4749>

We reviewed these suggestions carefully but did not see how we could incorporate them into this paper. We’ll keep these references for potential future work though.
5. The problem that author is trying to address in this paper is not clear yet, (clearer motivation for study is required)

We adjusted the introduction to more clearly outline the problem we are addressing.

6. Add the contribution of this study and study organization towards end of Introduction Section

We added more details about the contribution of our study to the literature and the structure of our approach.

7. Table 1 title need to be revised

We adjusted the title to increase clarity.

8. In 4-5- lines, authors should summarize the literature gaps identified from the review of the before the methodology section

We added some additional information explaining relevant gaps in the literature and how our approach contributes to the literature about human-wildlife conflict reporting.

9. Author should provide the mathematical illustration for the evaluation

We elaborated on our rationale for using path analysis and added a citation that provides more information about how such models are handled mathematically.

10. Services of language expert are required

We reviewed the English throughout the manuscript.
11. Image quality needs to be improved

We increased the resolution of images, ensuring each had a dpi of 300 and that the dimensions aligned with PLOS ONE’s figure formatting guidelines.
12. Ensure that all Figures are properly labeled and referenced

We reviewed and reined figure captions and labelling**.**

**E.g.,**

13. Authors should include the future work of this study

We added more information about how our approach could be applied in other contexts of human-wildlife conflict and elaborated on potential future work.

**Reviewer #2:**

1- The title needs to be simplified further.

We simplified the title while ensuring it remains informative.

2- The abstract did not indicate the criteria adopted in stating the efficiency of this proposal.

We aren’t sure what is meant by this comment, but we reassessed the adherence of our abstract to contain the conventional information (IMRaD) and edited it to improve clarity and flow.

3- Add a paragraph indicating the structure of the research with all its sections. It is placed at the end of the introduction

We adjusted some phrasing and more clearly illustrated the structure of our approach but did not add an additional paragraph, preferring to keep additional details about our approach in the methods section which is consistent with published examples in PLOS ONE.

4- Explain the related works and the negatives they suffered from and in which this manuscript excels.

We elaborated on the gaps in the literature and limitations of past papers while more clearly explaining how this manuscript addresses these gaps and limitations.

5- The work algorithm needs to be simplified and explained in direct and clear points.

We reviewed the methods for clarity and completeness and added several details.

6-Where are the conclusions and future work?

We increased discussion about our results and the implications of our results with respect to Edmonton’s coyote education campaign and other human-wildlife conflict management contexts and included more explicit details about potential future work.

**Reviewer #3:** Clarify "Unsure" Responses: It would be helpful to provide more details on how you handled "unsure" responses in the path model. This would add transparency and ensure readers fully understand the treatment of this data.
We added information to the methods to explain how the WLSMV estimator handles missing values via pairwise deletion.

Expand Practical Applications: The discussion could benefit from more details on how the findings can be applied in real-world urban wildlife management, particularly for educational campaigns and improving reporting systems.
We added additional detail in the Management implications section explaining how our approach could be tailored to inform education, refine reporting, and support management in other human-wildlife interaction contexts.

Explain Unsupported Hypotheses: The reasoning behind some of the unsupported hypotheses, especially the unexpected relationship between knowledge of food conditioning and risk perceptions, needs further clarification to help readers understand these results.
In the discussion, we further explained our unsupported hypotheses and why we may have seen these results.

Small Effect Sizes: Although the small effect sizes are acknowledged, they may limit the practical significance of the findings. It might be useful to discuss this limitation in more depth.
We adjusted phrasing to emphasize the small effect sizes we observed and offered some potential reasons for them.

Sample Bias: The sample is somewhat biased toward older, English-speaking respondents. While this is noted, it could impact how generalizable the results are, so it's worth discussing this a bit further.
We included more details about how the bias in our sample may affect the generalizability of our results.

Limited Generalizability: Since the study is focused on Edmonton, the findings may not directly apply to other cities with different urban dynamics or wildlife. This could be emphasized more clearly in the limitations.

Similarly to above, we better explained how our focus on Edmonton presents limitations for generalizing our results to other municipalities or species. We also included more explanation about how our approach, rather than our exact results, can be applied to human-wildlife conflict reporting and management in other locations and contexts.

**Reviewer #4:** - Based on previous similar works, the authors identified the unsolved challenges and proposed a study to examine how they could less influence the problem-based learning process.
- The paper is well-structured and documented.
- An overview on related works, some from recent years, is presented. It contains studies from specific literature.
- The theoretical background is well explained and properly used.
Thank you for the positive feedback.
Areas of Improvements
- The study has some limitations, which supposed to be identified by the authors and clearly mentioned.

We changed some phrasing to be more explicit about our limitations and their implications for interpreting our results.

- Corrections should be made related to the use of English language.

We reviewed the entire manuscript to identify grammatical errors and awkward passages and adjusted several sentences to increase clarity.

**Reviewer #5:** 
Strengths:
1. Large sample size provides good statistical power
2. Use of path analysis allows examination of direct and indirect effects
3. Clear rationale and relevance for urban wildlife management
4. Acknowledgment of limitations
Thank you for these comments; we appreciate them.
Suggestions for improvement:
1. Provide more details on the survey development process and pilot testing

We added some details to the methods to explain how we developed the survey. It was reviewed by several individuals at the City of Edmonton, but there was no pilot version, which we now explain.

2. Further discuss potential reasons for small effect sizes

We further explained why we may have seen small effect sizes.

3. Expand on management implications, particularly regarding education/communication strategies

Thank you for this suggestion. We adjusted some phrasing to improve clarity and further explained how our results can be applied to Edmonton’s coyote education campaign and how our approach can be used in other locations and contexts.

4. Consider adding a figure showing the distribution of responses to the two reporting scenarios
We appreciated this suggestion and added a bar plot as Figure 2 showing the distribution of responses to the scenarios. The final path model results are now shown in Fig 3.

Overall, this is a well-conducted study that provides useful insights for improving citizen reporting of urban coyote encounters. The findings have practical implications for wildlife managers and public education efforts.

Thank you.
