# Supplementary material for: Knowledge increases informative reporting by the public about urban coyotes
Source: PLoS One. 2025 May 9;20(5):e0307728. doi: 10.1371/journal.pone.0307728 (PMC12063906; doi:10.1371/journal.pone.0307728)
Supplement: Attachment — Submitted filename: Response_to_Reviewers_auresp_2.docx [file pone.0307728.s004.docx]

02 April 2025

Dear Editor,

Thank you for the invitation to revise and resubmit the manuscript “Knowledge increased informative reporting by the public about urban coyotes”. We appreciate the feedback from you and the reviewers and have edited our manuscript accordingly.

The main changes we made to the manuscript were to run a second version of the path model using only the response to our conflict-prone coyote scenario as our response variable, re-organize information to include the additional model and improve clarity and flow, and expand and refine our discussion in light of the results of the additional model.

At the end of this letter, we copied the reviewers’ comments (in black) and followed each comment with a description of how we addressed it (in blue + a screenshot showing the changes). Thanks for your attention to our manuscript; we look forward to hearing from you.

Sincerely,

Abby L. Keller

**Reviewer #4:** Authors have incorporated the mentioned changes as raised in earlier version. Overall, the paper is good, organised, well written and the content is significant for the field related to the problem-based learning approaches in computer science.

Thank you.

**Reviewer #6:** This study uses a thorough social survey and complimentary statistical analysis to evaluate the effects of personal experience, knowledge, and demographic factors on the likelihood of efficient reporting of human-coyote conflict. This is a widely relevant topic as coyote populations are flourishing in urban areas across the continent, and community reporting of wildlife conflict is becoming increasingly common worldwide. The paper's methods are fully appropriate for the questions being asked, and I believe the statistical approach to be sound. I was impressed with the survey response rate.

Thank you.

The major statistical limitation is that the manner of calculating reporting effectiveness left very little variation in the model's primary response variable, leading to small effect sizes and somewhat tenuous interpretation of the outputs. Did the authors try and perform the same analysis on other versions of the response variable, like the raw score of likelihood to report a conflict sighting (without subtracting the benign score)? I am curious how this would change the results or if more resolution could be gained.

Thank you for this suggestion. Following this, we ran another version of the model with the raw score of likelihood to report a benign sighting as the response variable. We chose to use the benign sighting score rather than the conflict-prone score because we felt understanding what factors predict anticipated reporting of the benign scenario could better support the City of Edmonton’s goal of reducing the volume of reports received about benign sightings of coyotes. We placed this new model before our original model throughout the text for the sake of flow and clarity with respect to our original model.

The results of this model provided further support for and helped us to refine our recommendations for a coyote education campaign aimed at reducing reports of benign sightings and encouraging reports of conflict-prone coyotes.

The following are a few examples of changes we made to the discussion to incorporate the results of the new model:

As mentioned by other reviewers, the embedded figures were low resolution but that may have been addressed with additional file uploads. The manuscript's writing/grammar showed no issues, and it is overall well-written and understandable.

Line comments:
275: Remove the word 'greater'

Done.

303-305: Was this result presented in the paper? If not, some support is needed

Thanks for catching this. We did not explicitly present this in the results, but we added a reference to Appendix A, where the responses to the risk items are presented in detail.
